# Evaluation of the profile of alopecia areata and the prevalence of thyroid function test abnormalities and serum autoantibodies in Iranian patients

**DOI:** 10.1186/1471-5945-5-11

**Published:** 2005-10-31

**Authors:** Hassan Seyrafi, Maryam Akhiani, Hamed Abbasi, Sahar Mirpour, Ali Gholamrezanezhad

**Affiliations:** 1 Department of Dermatology. Tehran University of medical sciences. Tehran. Iran

## Abstract

**Background:**

The study aimed at evaluating the prevalence of thyroid function abnormalities in patients with alopecia areata (AA) and its association with other autoimmune diseases and various autoimmune antibodies.

**Method:**

We retrospectively analyzed medical records of 123 patients with AA. The main site of involvement, pattern, and extent of alopecia as well as presence of the similar disease in first-degree family members and serologic status of patients were recorded.

**Results:**

Participating in the study were 57 males and 66 females (6 to 59 years old). In the majority of patients (69.9%) the disease was manifested in the first two decades of life. Patients with family members having alopecia were recorded in 24.4%. Thyroid function abnormalities were found in 8.9% of patients. Positive autoimmune antibodies were associated with AA in 51.4% of patients with no significant association between the severity and duration of disease and presence of these antibodies.

**Conclusion:**

The incidence of positive auto-immune antibodies in Iranian patients is higher than previous reports. Concerning the female:male ratio, thyroid function tests and the prevalence of alopecia in first-degree relatives, our results are compatible with previous data obtained from different ethnic populations. Previous reports documented that a greater severity and longer duration of AA were seen in the early onset forms; however our result are relatively different which could be explained by differences in genetic factors.

## Background

Alopecia areata (AA) is a non-scarring hair disorder, the etiology of which is minimally understood. Since human hair has an important communicational role and also because it is predominantly a disease of the youth, this disorder is able to cause significant psychological distress. Therefore, it would be extremely grateful to find appropriate measures to overcome this relatively stressful disorder. This aim is only when achieved that the main underlying causes of the disorder have been discovered.

Although many different pathogenic causes have been proposed, the determination of the exact underlying etiology of AA is extremely problematic. In fact, these difficulties are in part due to variable extent of the disease and the heterogonous and poorly defined nature of the patients studied. Of the numerous pathogenic processes which have been proposed as the underlying pathogenic causes of the AA, immunological, environmental, psychological, and genetic factors [1,2] are the most powerful explanations, but the relative significance of each is not completely known. For example, the genetic basis is explained by a higher familial occurrence, with a positive family history in 10–42% of patients in different populations [3]. There are also lots of data concerning the contribution of autoimmune processes in the pathogenesis of AA and in fact these evidences are more convincing [4]. The association of AA with other auto-immune processes, such as auto-immune thyroiditis and diabetes mellitus has been widely reported and has been considered as a potent indicator of the contribution of auto-immunity in the pathogenesis of the AA [5]. Although all these evidences suggest that the hair can be considered a target organ for autoimmune processes, presence of remarkable data concerning the contribution of psychological, environmental and genetic predisposing factors make it difficult to determine the exact cause of the disorder.

Also there is a lack of agreement on the overall prevalence of thyroid disease and thyroid function abnormalities in alopecia areata [6] and the prevalence of thyroid disease in patients with alopecia areata in previous reports varies from 8 to 28% [7]. Unfortunately, no study is available from the Iranian subcontinent.

The aim of our study was to evaluate the frequency of thyroid function abnormalities, antithyroid auto-antibodies and few other well-known autoimmune antibodies [antinuclear antibody (ANA), anti-smooth muscle antibody (SMA), anti-parietal cell antibody (PCA), anti-thyroglobulin antibodies (anti-Tg)] in Iranian patients affected by AA. Moreover, we intended to assess the prevalence of AA between the first degree relatives of our patients.

## Methods

The study was carried out at the dermatology department (Tehran University of medical sciences) between February 2002 and July 2004. The data were collected retrospectively and systematically in a pre-established questionnaire. AA was diagnosed according to the definition of Olsen et al. [8]. The extent of hair loss was classified as < 50% (S1–S2) involvement, 50–99% (S3–S4) involvement, alopecia totalis (AT), and alopecia universalis (AU) at the time of presentation. All patients with AA were entered in the analysis. During the data collection, the main site of involvement, pattern, and extent of alopecia were recorded. Also a physical examination directed toward signs of other systemic or autoimmune diseases and the history of AA in first degree relatives were stated.

The data of serologic status [ANA, SMA, PCA, anti-Tg, and routine thyroid function tests (including Free T3, Free T4, and TSH)] were collected from the medical records of patients. The serum level of SMA, ANA and PCA (positive titre ≥1:80) were measured by indirect immunofluorescence (IIF) according to the standard protocols (Iason Labormedizin, Graz, Austria). Free T3 (FT3, normal range: 3–7.5 pmol/l), TSH (normal range: 0.5–3.5 mU/l), and anti-Tg (positive titer > 1:100) were measured by the chemiluminesence fluorescence method (Bayer Diagnostics, Leverkusen, Germany).

### Statistical analysis

The associations were analyzed by chi-square, Fischer bicaudal exact test and T test. A probability of less than 0.05 was considered significant. SPSS for Windows (Release 11.5.0) was used for statistical analysis.

## Results

A total of 123 patients with Alopecia disorders, including 57 (46.3%) males and 66 (53.7%) females were entered in our analysis (female: male ratio = 1.15:1). Of these, 57 patients (46.3%) had AT, 12 patients (9.8%) AU and 54 patients (43.9%) patchy alopecias. The age of patients at the onset of the disease had a wide range from 6 to 59 years (24.05 ± 9.98, mean ± SD). The peak age at the onset of the disease for either sex was 15 to 25 years, constituting 45.5% of the whole population of the patients. 86 patients (69.9%) experienced their first episode of AA before 20 years old. Although involving with the severe forms of the disease was seen more frequently in those patients who had early onset alopecias, but considering the severity and the duration of the disease no significant difference was found between the different age groups of patients.

Involvement of the family members with AA was found in 30 of 123 patients (24.4%). Abnormalities of thyroid function were detected in 11 (8.9%) patients included 7 (63.6%) male and 4 (36.4%) female. Of these, 3 patients (2.4%) had T3, 4 patients (3.2%) T4 and 10 patients (8.2%) TSH abnormalities. Presence of auto-immune antibodies (ANA, SMA, Anti-Tg and PCA) was established in 62 patients (51.4%). Of the 123 patients investigated for Anti-Tg, 36 patients (29.3%) had positive titer, including 27 men (22%) and 9 women (7.3%). Of these, positive titers were detected in 16 patients with the patchy form of the disease (13%) and 20 patients with AU (16.3%) (Table 1). Although positive titer was observed in 22 patients (21.1%) affected with AA before 20 years, but there was no statistically significant difference between the positivity of Anti-Tg test and the age of patients at the onset of the disease (Table 2). A higher proportion of patients (18.2%) with a longer duration of disease (>5 years) compared with patients with length of disease less than 1 year (1.9%) have positive anti-Tg titer, but differences did not attain statistical significance. Of 19 (15.5%) patients with positive PCA titer, 13 patients (10.8%) presented with AU and 6 (4.9%) with patchy forms but there was no significant association between the severity of disease and positivity of PCA. Of 19 patients who have positive PCA titer, 11 patients (6.9%) were afflicted longer than 5 years. There were also no significant differences between duration of disease and presenting positive anti-parietal antibody. 5 (4.8%) of 123 patients presented with SMA, all of them were between 15 to 25 years and the onset of disease was before 10 years old (Table 3). Two (1.8%) women in the patchy form group were seropositive for ANA. Both were involved before 20 years old and afflicted by disease for more than 5 years (Table 4).

**Table 1:** The frequency of positive anti-Tg antibody among the different forms of AA.

**Anti-Tg Antibody/Clinical form**	Positive		Negative	
	
	n	%	n	%
**Patchy**	16	13	14	11.4
**AT**	0	0	57	46.3
**AU**	20	16.3	16	13
**Total**	36	29.3	87	70.7

**Table 2:** The frequency of positive anti-Tg antibody among the different age groups.

**Anti-Tg Antibody/Age group**	Positive		Negative	
	
	n	%	n	%
**5–14**	5	4.1	13	10.5
**15–24**	20	16.2	33	26.9
**25–34**	6	4.9	21	17
**35–44**	5	4.1	11	9
**>44**	0	0	9	7.3
**Total**	36	29.3	87	70.7

**Table 3:** The frequency of positive anti-smooth muscle antibody among the different age groups.

**Anti SM Ab/Age group**	Positive		Negative	
	
	n	%	n	%
**5–14**	0	0	21	17
**15–24**	6	4	38	31
**25–34**	0	0	26	21.1
**35–44**	0	0	21	17
**>44**	0	0	11	9
**Total**	5	4.8	118	95.1

**Table 4:** The frequency of positive ANA among the different age groups.

**ANA/Age group**	Positive		Negative	
	
	n	%	n	%
**5–14**	0	0	18	16.2
**15–24**	1	0.9	49	44.1
**25–34**	0	0	26	23.4
**35–44**	0	0	13	11.8
**>44**	1	0.9	3	2.7
**Total**	2	1.8	109	98.2

## Discussion

Alopecia is an ancient disease and was known to Egyptians even before Christ [9]. Despite its long history, our knowledge is actually limited. Generally, significant differences have been identified in the profile of the disease among different societies [10].

Previous studies have revealed that AA affects both sexes equally [11,12] with females slightly more predominanated [3,13,14]; Similar findings have been obtained in our study with slight predominance of the females (female:male ratio = 1.15:1).

AA may begin as early as the fourth month of the life [15] or as late as in the late seventies [12]. Our findings were not very different and the youngest patient in our group of patients was a 6 years and the oldest was 59. The prevalence of AA presenting before 20 years was reported previously to be between 27–44% [16], whereas the result in the current study was interestingly higher (69.9%). This disparity is difficult to explain and could be due to racial and genetic factors.

AA has been considered as an auto-immune disease, due to an aberrant T cell response against hair follicle self-antigens [17]. This auto-immune etiology has been also proposed on the basis of its association with various auto-immune diseases, the presence of auto-antibodies and various underlying immunologic abnormalities in the affected sites of these patients [10].

One of the main associations is with thyroid abnormalities [5]. The incidence of thyroid disease has varied from 8 to 28% in patients with AA [16]. Milgraum et al. also found an apparent association between thyroid disease and AA [4]. Subsequently Lewinski et al confirmed the frequent co-existence of AA and thyroid abnormalities [18]. Conversely, in 1994 Puavilai et al. estimated that the prevalence of thyroid disease is relatively low (7.2%) and they were not statistically different from patients with AA and control group [7]. In our study, 8.9% of patients had abnormal thyroid function tests, which relatively correlate with previous reports. We should note that the prevalence of thyroid disease in the Iranian normal population is 2.97% [19], hence our patients show higher incidence of thyroid diseases in comparison with normal individuals. Many reports found significant associations between alopecia areata and autoimmune endocrinopathies [5], but other studies do not confirm this [20]. In a report of large number of cases from North America [3], auto-immune diseases were closely associated with AA in 17.1% of patients. However, in the study of Vinod et al in 1996, the corresponding features were much lower (5%) [16].

In previous studies SMA and PCA were found in 34.6% and 42.3% of the patients, respectively, followed by antithyroglobulin antibody in 2.8% [21]. In our study, these auto-immune antibodies were associated with AA in 15.5% and 4.8% of patients, which are much lower than the previous report of Kumar et al. These authors have themselves also interested in their results and mentioned that AA in India is associated more often with antismooth muscle and antiparietal cell antibodies.

Generally the frequency of autoantibodies in Iranian patients (51.4%) is higher than the other populations [3,16]. It is next to impossible to offer an adequate explanation of the real causes of these differences and may partly be attributed to racial and genetic factors.

It is well-known that various kinds of auto-antibodies are found in each population of healthy individuals. The limitation that forced itself upon our research study was the fact that because of retrospective nature, we did not posses any control group in order to compare the results obtained from the study of antibodies; however, it must be admitted that a report concerning the rate of antithyroglobulin and ANA antibodies in normal Iranian population exists [19]. The result of this study has reported the rate of anti-Tg antibody and ANA, 16.2% and 1.7% of the normal Iranian population, respectively. We have made a comparison of the anti-Tg and ANA rate in our patients with the above result. In our study the prevalence of positive anti-Tg antibodies in patients was higher than normal Iranian population (29.8% versus 16.2%), but the frequency of ANA titer is relatively equal in our patients and normal individuals (1.8% versus 1.7%). On the other hand, the prevalence of these antibodies in our patients is not consistent with the previous study of Okamoto et al [22], in which the prevalence of positive ANA in the patients afflicted with AA was remarkably higher than our patients (53% versus 1.8%). Over all, on account of genetic factors, the prevalence of auto-antibodies is not similar in the different societies, even in the normal population. For example the positive titer of ANA in the normal Iranian population is 1.8%, while it was estimated that in the normal Japanese it is about 15% [22]. This difference emphasize on the fact that we could not compare the results of different communities. Unfortunately, we could not find any information about the value of SMA and PCA in normal populations.

Family members having AA has been reported in 10 to 20% of patients [12-14]. Our figure of 24.4% is close to the upper limit of the above reports and this higher prevalence of the disease in the first degree relatives of the patients as compared to the previous reports of the other communities, emphasizes again on the importance of the genetic factors in our population. However, it should be also considered that the patients in the clinic of a University Hospital may have disease more severe than the average and this may be the reason for the high frequency of a positive family history. On the other hand, in previous publications, the highest family history rates have often been in small series and bias in ascertainment of the cases is often the reason for this.

## Competing interests

The author(s) declared that have no competing interest.

## Authors' contributions

HS and MA participated in the design of the study and supervised the study progress. HA performed the data collection. SM and AG participated in the statistical analysis and drafted the manuscript. All authors read and approved the final manuscript.

## Pre-publication history

The pre-publication history for this paper can be accessed here:


